# Sloth Hair as a Novel Source of Fungi with Potent Anti-Parasitic, Anti-Cancer and Anti-Bacterial Bioactivity

**DOI:** 10.1371/journal.pone.0084549

**Published:** 2014-01-15

**Authors:** Sarah Higginbotham, Weng Ruh Wong, Roger G. Linington, Carmenza Spadafora, Liliana Iturrado, A. Elizabeth Arnold

**Affiliations:** 1 Smithsonian Tropical Research Institute, Panama, Republic of Panama; 2 Department of Chemistry and Biochemistry, University of California Santa Cruz, Santa Cruz, California, United States of America; 3 Instituto de Investigaciones Científicas y Servicios de Alta Tecnología, Panama, Republic of Panama; 4 School of Plant Sciences, University of Arizona, Tucson, Arizona, United States of America; Louisiana State University, United States of America

## Abstract

The extraordinary biological diversity of tropical forests harbors a rich chemical diversity with enormous potential as a source of novel bioactive compounds. Of particular interest are new environments for microbial discovery. Sloths – arboreal mammals commonly found in the lowland forests of Panama – carry a wide variety of micro- and macro-organisms on their coarse outer hair. Here we report for the first time the isolation of diverse and bioactive strains of fungi from sloth hair, and their taxonomic placement. Eighty-four isolates of fungi were obtained in culture from the surface of hair that was collected from living three-toed sloths (*Bradypus variegatus*, Bradypodidae) in Soberanía National Park, Republic of Panama. Phylogenetic analyses revealed a diverse group of Ascomycota belonging to 28 distinct operational taxonomic units (OTUs), several of which are divergent from previously known taxa. Seventy-four isolates were cultivated in liquid broth and crude extracts were tested for bioactivity *in vitro*. We found a broad range of activities against strains of the parasites that cause malaria (*Plasmodium falciparum*) and Chagas disease (*Trypanosoma cruzi*), and against the human breast cancer cell line MCF-7. Fifty fungal extracts were tested for antibacterial activity in a new antibiotic profile screen called BioMAP; of these, 20 were active against at least one bacterial strain, and one had an unusual pattern of bioactivity against Gram-negative bacteria that suggests a potentially new mode of action. Together our results reveal the importance of exploring novel environments for bioactive fungi, and demonstrate for the first time the taxonomic composition and bioactivity of fungi from sloth hair.

## Introduction

Despite vast increases in spending on international healthcare over the last 20 years, communicable diseases continue to represent an enormous burden to global health [1]. Chronic infectious illnesses such as malaria and diverse neglected tropical diseases (NTDs) affect millions of people each year, mainly women and children in developing countries [2]. The rapid spread of antibiotic resistance has decreased the arsenal available to treat many infectious diseases, with recent emergence of pandrug resistance yielding illnesses that are recalcitrant to treatment with any known drug [3]. Concurrently non-communicable diseases are rising in incidence compared to communicable diseases: in 2008 cancer was responsible for about 7.8 million deaths (about 13% of all deaths worldwide; [4]).

Natural products represent one of the most significant sources of new drugs today. Approximately 50% of all medicines introduced between 1981 and 2006 were of natural product origin [5], with an additional 34 natural product-based drugs launched between 1997 and 2007 [6]. Since the discovery of penicillin over 80 years ago, fungi have contributed enormously to natural product drug discovery, providing a host of invaluable medicines including the β-lactam antibiotics, griseofulvin, cyclosporine, fusidic acid, and lovastatin [7]. However, in recent years the hit-rate for bioactivity from fungi has slowed, possibly pointing to the impending exhaustion of ‘low hanging fruit’ – the easily and frequently accessed fungi such as soil microfungi – as sources of new bioactive metabolites.

Conservative estimates suggest that the total number of fungal species in existence exceeds 5 million, yet fewer than 100,000 fungal species have been described [8] [9]. This suggests that exploring new environments that may be home to previously undescribed fungi could be extremely productive. For example, recent examination of tropical fungal endophytes has described tropical leaves as a ‘biodiversity hotspot’ [10] [11] [12] that is proving fruitful as a source of new bioactive compounds (see [13] for a review).

Gloer [14] proposed that production of secondary metabolites by fungi may be influenced by the selective pressures imparted by other organisms. This suggests that environments where many different species exist in close proximity may have particular potential as sources of bioactive metabolites. One such unexplored environment is the fungal microbiome associated with the coarse, sponge-like hair of the three-toed sloth (*Bradypus variegatus*), an arboreal mammal commonly found in the lowland tropical forests of Central America.

The coat of three-toed sloths consists of two distinct layers: an inner layer of fine, soft hair close to the skin, and an outer layer of coarse hairs that are oval in cross section and are approximately 0.4 mm wide [15]. These outer hairs carry transverse cracks that are home to an apparently ubiquitous green alga, most commonly of the genus *Trichophilus* [16]. The alga is popularly thought to provide camouflage for sloths against the mottled background of their habitat, but this has not been confirmed with data. Suutari *et al.* [16] suggested that exopolymeric substances produced by the alga might encourage the growth of beneficial bacteria. Sloth hair is commonly home to cyanobacteria and diatoms, as well as a variety of macro-organisms (e.g., cockroaches, roundworms, and moth larvae; [16]). However, little is known about communities of fungi on sloth hair.

In an effort to uncover new sources of drugs for treating vector-borne diseases, cancer, and bacterial infections, we used a culture-based approach to examine fungal communities associated with the coarse, outer hair of *Bradypus variegatus* in Panama. Here we report the isolation of fungi with bioactivity against *Trypanosoma cruzi*, the causal agent of Chagas disease; *Plasmodium falciparum*, the causal agent of malaria; the human breast cancer cell line MCF-7; and a range of Gram-negative and Gram-positive human pathogenic bacteria. Phylogenetic analyses were used to identify these fungi at a finer and more robust level than is possible using typical database-matching methods alone. Our results suggest that sloth hair is an interesting new source of important bioactive fungi with much scope for exploration of the five other extant sloth species found across the neotropics.

## Materials and Methods

Approval for sampling of sloth hair was obtained from the Institutional Animal Care and Use Committee (IACUC) of the Smithsonian Tropical Research Institute and collection permits were obtained from Panama's Autoridad Nacional del Ambiente (ANAM). A single sample of coarse outer hair was collected from around the lower back of each of nine living three-toed sloths encountered in February 2011 along Pipeline Road in Soberanía National Park, Republic of Panamá (N 9°9′, W 79°44′; [17]). Hair samples were placed in sterile Falcon tubes half filled with silica gel and stored at ambient temperature until processed.

### Isolation and cultivation of fungi

Each hair was rinsed with sterile water to remove debris and loosely associated microorganisms. Under aseptic conditions hairs were cut into pieces approximately 2.5 cm long and placed on the surface of potato dextrose agar (PDA) or 2% malt extract agar (MEA) in sterile Petri plates. Plates were sealed with Parafilm, incubated at room temperature, and checked for new fungal growth daily for 2 weeks. Hyphae were cut aseptically from each plate and transferred to axenic culture on the same medium. Isolates were stored as living vouchers at room temperature (i.e., as agar plugs with mycelium in sterile distilled water) and have been archived in the collection of the International Cooperative Biodiversity Group (ICBG) at the Smithsonian Tropical Research Institute in Panama (accessions available on request).

### Fungal identification

Total genomic DNA was extracted from fresh cultures of fungi according to Arnold & Lutzoni [11]. The nuclear ribosomal internal transcribed spacers and 5.8S gene (ITSrDNA) and an adjacent portion of the nuclear ribosomal large subunit (LSUrDNA) were amplified as a single fragment using primers ITS1F or ITS5 and LR3 following Hoffman *et al.* [18]. PCR products were visualized using SYBR green following electrophoresis on a 1% agarose gel and submitted to the University of Arizona Genetics Core for cleanup, normalization, and bidirectional Sanger sequencing. Sequences were assembled automatically and bases called using *phred* and *phrap* [19] [20] with orchestration by Mesquite [21], followed by manual editing in Sequencher 4.5 (GeneCodes Corp.). Sequences were compared against the NCBI non-redundant database using BLASTn [22] to estimate taxonomic placement and were submitted to GenBank under accession nos. KF746076-KF746159 (Table S1).

Because identification based only on BLAST matches must be treated with caution (e.g., [23]) we used phylogenetic analyses to provide stronger inference regarding taxonomic affiliation. Sequence data for 84 fungal isolates were loaded as one group into Mesquite [21] and aligned by MUSCLE [24]. The resulting alignment, which identified clusters of easily alignable sequences, was inappropriate for a single phylogenetic analysis due to the prevalence of ambiguous and unalignable regions. Sequences that aligned coherently to one another were partitioned into groups of apparently closely related strains. Resulting groups were then analyzed as separate data sets as follows: All sequences in each group were compared against the NCBI database using BLASTn. The top 50 hits for each sequence were downloaded and filtered to remove (1) redundant sequences, and (2) sequences from potentially mis-identified strains and unvouchered specimens (see [23]). At least one sequence from a reliable culture collection was included in each data set, and one or two outgroups were selected from species closely related to named sequences in the dataset (chosen by literature review).

Each resulting data set was aligned separately in MUSCLE. Sequences in each alignment were trimmed to consistent starting and ending points, and alignment quality was verified by visual inspection in Mesquite. All alignments were submitted to TreeBASE (http://purl.org/phylo/treebase/phylows/study/TB2:S14945). Models of evolution were inferred using jModeltest [25] [26]. Phylogenetic trees were inferred by maximum likelihood in GARLI [27], with support determined by 100 bootstrap replicates implemented in the CIPRES web portal [28], and using Bayesian methods in MrBayes (accessed via CIPRES [28]). For the latter, analyses consisted of 5 million generations, with four chains, random starting trees, and sampling every 1000th tree. Completeness was assessed based on asymptotes of the –ln li values and evaluation of standard deviations of split frequencies. Posterior probabilities provided inferentially independent values to support the topological relationships and associated support values from maximum likelihood analyses. Results of each phylogenetic analysis were compared against the existing literature for each taxonomic group to ensure that relationships of known taxa were appropriate, thus providing confidence with regard to our placement of sloth hair fungi.

### Preparation of fungal extracts

A single agar plug of each strain selected for further analysis was transferred under sterile conditions to a fresh plate of 2% MEA and incubated on the benchtop at room temperature until approximately 50% of the plate was covered with mycelial growth. Fifteen agar plugs (each 5 mm in diameter) were cut with a sterile cork borer under sterile conditions and transferred to flasks containing 37 ml of 2% malt extract broth (MEB). Flasks were incubated on an orbital shaker (28°C, 125 rpm) for 2 weeks.

Liquid cultures were mixed with an equal volume of ethyl acetate and blended for 2 minutes at 9000 rpm with a Polytron (Lauda-Brinkmann, Delran, NJ, USA). Fungal biomass was removed by vacuum filtration through Whatman filter paper (#1) and the filtrate was extracted twice with a 1∶1 volume of ethyl acetate. The aqueous layer was discarded and the organic layer was dried and stored at −80°C.

### Bioassays

Crude organic extracts of fungal cultures were used in bioassays against focal strains of the causal agents of malaria (*Plasmodium falciparum*) and Chagas disease (*Trypanosoma cruzi*), and against breast cancer cell line MCF-7, as described by Higginbotham *et al*. [29]. Bioactivity of extracts, which were diluted in DMSO (10 µg/ml), was measured as percent inhibition of growth (% IG) compared to the negative control (DMSO with no extract; 0% IG).

### BioMAP analyses

An antibiotic activity profile screen, BioMAP (Anti*bio*tic *m*ode of *a*ction *p*rofile; [30]), was used to test activity of 50 crude organic extracts against 15 human pathogenic bacteria. Extracts with potent bioactivity were serially diluted (16 2-fold dilutions) and re-screened against the same panel of bacteria. Growth curves were plotted by recording OD_600_ values each hour for 24 hours and minimum inhibitory concentrations (MIC) were subsequently calculated for each extract. To establish concentration-independent bioactivity profiles for each extract, raw MIC values were normalized resulting in a range of values from 0 (inactive) to 1 (most bioactive). These activity profiles were then compared to profiles of known antibiotics belonging to all major structural classes. This method is effective in predicting the structural class of unknown antibiotic compounds from complex or crude mixtures [30].

## Results and Discussion

**Figure 1:**
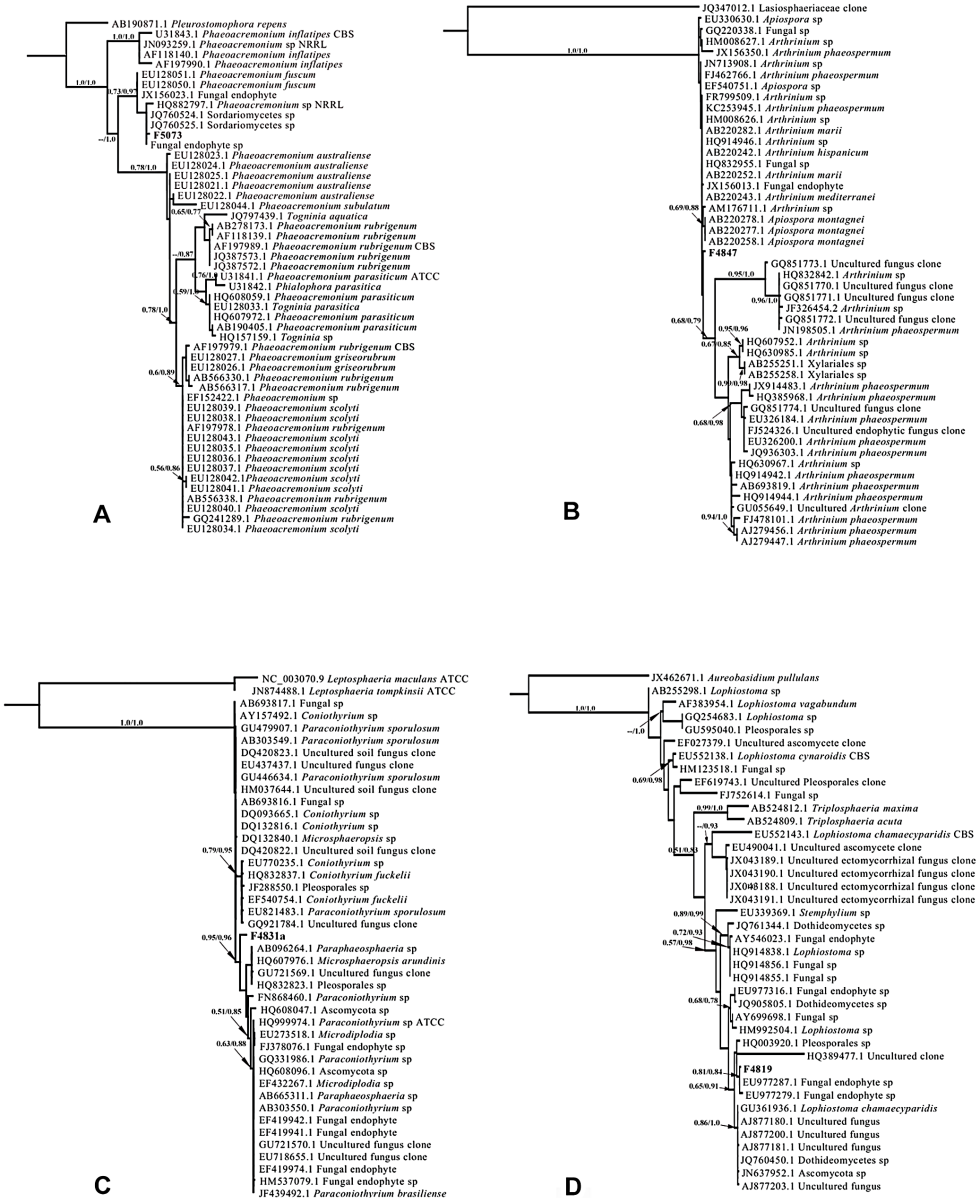
Results of maximum likelihood and Bayesian analyses of ITSrDNA data for fungi isolated in culture from coarse, outer hair of three-toed sloths in Soberanía National Park, Panama. Topology of each tree reflects ML analysis, and values above branches indicate ML bootstrap values and Bayesian posterior probabilities (>0.50 and >0.75, respectively). Outgroups and taxon sampling for each tree were validated by literature surveys (see methods). Taxonomic conclusions are presented in Table 1. Figure 1(A): Placement of F5073 in group 17; (B) F4847 in group 18; (C) F4831a in group 19; (D) F4819 in group 20; (E) F4801 in group 5; (F) F4806 in group 7; (G) F4886 in group 8; (H) F5071 in group 15; (I) F4812–F4816, F4830, F4831, F4845, F4852–F4856, F4860, F4873, F4882, F4883 and F4900–F4902 in group 1; (J) F4803, F4817, F4820, F4823, F4824, F4826, F4827, F4829, F4837, F4841, F4842, F4846, F4848, F4857, F4858, F4861, F4862, F4870, F4872, F4875, F4878, F4879, F4894–F4896, F4908, F4909, F5069 and F5074 in group 2; (K) F4818 and F4839 in group 10; (L) F4877, F4890 and F4897 in group 11; (M) F4828 and F4898 in group 12; (N) F4876 and F4881 in group 13; (O) F4863 and F4884 in group 6; (P) F4821, F4874, F4889 and F4913 in group 3; (Q) F4802, F4807, F4825, F4844, F4906 and F5068 in group 4; (R) F4850 and F4891 in group 9; (S) F4904 and F4905 in group 14; and (T) F5070 and F5072 in group 16.

A total of 84 fungal isolates was examined from the coarse outer hair of nine living individuals of the three-toed sloth (*Bradypus variegatus*) encountered along Pipeline Road in Soberanía National Park, Republic of Panama. Hair samples were transported to the lab in sterile Falcon tubes which had been half filled with silica gel, a desiccant that is effective for storing fungal tissue [31] [32]. Every piece of sloth hair placed on agar yielded multiple fungal isolates representing a variety of morphotypes. Phylogenetic analyses of axenic strains revealed a diverse group of fungi, some of which appear to be novel relative to previously observed or sequenced taxa (Table 1; Figure 1). Many of these isolates display bioactivity *in vitro* against parasites that cause malaria and Chagas disease, breast cancer cells, and both Gram-positive and Gram-negative human pathogenic bacteria (Table 2; Table 3).

**Table 1:** Eighty-four fungal isolates from the coarse outer hair of nine individuals sloths (*B. varieagatus*), their top BLAST matches, and maximum identity value from BLAST searches; group ID and tree (Fig. 1) revealing phylogenetic placement; and taxonomic placement based on phylogenetic analyses at the family (order) and genus levels (Fig. 1).

Fungus ID	Sloth hair Sample #	Top BLAST Hit, (Accession Number)	Max ID (%)	Group ID	Tree	Family (Order)	Genus	OTU
F4847	S006	Fungal sp., (HQ832955.1)¥	99	18	B	Apiosporaceae (Incertae sedis)	*Arthrinium*	S*
F4821	S007	*Bionectria* cf. *ochroleuca*, (EU552110.1)¥	99	3	P	Bionectriaceae (Hypocreales)	*Bionectria*	I
F4889	S008	*Bionectria* cf. *ochroleuca*, (EU552110.1)¥	99	3	P	Bionectriaceae (Hypocreales)	*Bionectria*	I
F4913	S009	*Bionectria* cf. *ochroleuca*, (EU552110.1)¥	99	3	P	Bionectriaceae (Hypocreales)	*Bionectria*	I
F4874	S005	*Bionectria pityrodes*, (JQ411387.1)¥	99	3	P	Bionectriaceae (Hypocreales)	*Bionectria*	W*
F4806	S008	*Colletotrichum* sp., (DQ300348.1)¥	99	7	F	Glomerellaceae (Glomerellales)	*Colletotrichum*	R*
F4839	S002	Ascomycota sp., (HQ608154.1) [58]	99	10	K	Valsaceae (Diaporthales)	*Cytospora*	D
F4818	S002	Sordariomycetes sp., (JX174122.1)¥	99	10	K	Valsaceae (Diaporthales)	*Cytospora*	D
F4801	S003	*Endomelanconiopsis endophytica*, (GQ469968.1)¥	100	5	E	Botryosphaeriaceae (Botryosphaeriales)	*Endomelanconiopsis*	U*
F5071	S003	Fungal sp., (FJ612981.1) [23]	99	15	H	Xylariaceae (Xylariales)	*Entonaema*	N*
F4876	S006	Fungal sp., (EU563515.1) [59]	99	13	N	Nectriaceae (Hypocreales)	*Fusarium*	X
F4828	S005	Fungal sp., (FJ612934.1) [23]	99	12	M	Nectriaceae (Hypocreales)	*Fusarium*	O*
F4898	S003	Fungal sp., (FJ613085.1) [23]	100	12	M	Nectriaceae (Hypocreales)	*Fusarium*	Y*
F4881	S009	*Fusarium concolor*, (GQ505763.1) [60]	99	13	N	Nectriaceae (Hypocreales)	*Fusarium*	X
F4897	S003	Uncultured soil fungus clone, (DQ420777.1) [35]	98	11	L	Nectriaceae (Hypocreales)	*Fusarium*	AA*
F4877	S003	Uncultured soil fungus clone, (DQ420802.1) [35]	99	11	L	Nectriaceae (Hypocreales)	*Fusarium*	K
F4890	S008	Uncultured soil fungus clone, (DQ420802.1) [35]	98	11	L	Nectriaceae (Hypocreales)	*Fusarium*	K
F4900	S004	Fungal sp., (FJ613088.1) [23]	100	1	I	Hypocreaceae (Hypocreales)	*Hypocrea*	E
F4812	S001	*Hypocrea jecorina*, (AF510497.1)¥	99	1	I	Hypocreaceae (Hypocreales)	*Hypocrea*	A
F4813	S001	*Hypocrea jecorina*, (EU280094.1) [61]	100	1	I	Hypocreaceae (Hypocreales)	*Hypocrea*	A
F4815	S001	*Hypocrea jecorina*, (EU280094.1) [61]	100	1	I	Hypocreaceae (Hypocreales)	*Hypocrea*	A
F4814	S001	*Hypocrea jecorina*, (JQ070073.1)¥	100	1	I	Hypocreaceae (Hypocreales)	*Hypocrea*	A
F4852	S001	*Hypocrea jecorina*, (JQ070073.1)¥	100	1	I	Hypocreaceae (Hypocreales)	*Hypocrea*	A
F4853	S001	*Hypocrea jecorina*, (JQ070073.1)¥	100	1	I	Hypocreaceae (Hypocreales)	*Hypocrea*	A
F4855	S001	*Hypocrea jecorina*, (JQ070073.1)¥	100	1	I	Hypocreaceae (Hypocreales)	*Hypocrea*	A
F4856	S001	*Hypocrea jecorina*, (JQ070073.1)¥	100	1	I	Hypocreaceae (Hypocreales)	*Hypocrea*	A
F4854	S001	*Hypocrea jecorina*, (JQ411369.1)¥	100	1	I	Hypocreaceae (Hypocreales)	*Hypocrea*	A
F4882	S009	*Hypocrea jecorina*, (JQ411369.1)¥	100	1	I	Hypocreaceae (Hypocreales)	*Hypocrea*	A
F4901	S004	*Hypocrea lixii*, (AY605743.1)¥	100	1	I	Hypocreaceae (Hypocreales)	*Hypocrea*	E
F4831	S005	*Hypocrea lixii*, (JF923806.1)¥	100	1	I	Hypocreaceae (Hypocreales)	*Hypocrea*	E
F4902	S005	*Hypocrea lixii*, (JF923806.1)¥	100	1	I	Hypocreaceae (Hypocreales)	*Hypocrea*	E
F4873	S005	*Hypocrea muroiana*, (JN943367.1) [62]	99	1	I	Hypocreaceae (Hypocreales)	*Hypocrea*	H
F4883	S006	*Hypocrea nigricans*, (JN943369.1) [62]	99	1	I	Hypocreaceae (Hypocreales)	*Hypocrea*	E
F4816	S001	*Trichoderma reesei*, (JQ979435.1)¥	100	1	I	Hypocreaceae (Hypocreales)	*Hypocrea*	A
F4830	S005	Uncultured Trichoderma clone, (JX317342.1)¥	100	1	I	Hypocreaceae (Hypocreales)	*Hypocrea*	H
F4845	S005	Uncultured Trichoderma clone, (JX317342.1)¥	99	1	I	Hypocreaceae (Hypocreales)	*Hypocrea*	H
F4860	S005	Uncultured Trichoderma clone, (JX317342.1)¥	99	1	I	Hypocreaceae (Hypocreales)	*Hypocrea*	H
F4825	S004	*Lasiodiplodia parva*, (GQ469961.1)¥	100	4	Q	Botryosphaeriaceae (Botryosphaeriales)	*Lasiodiplodia*	C
F4844	S004	*Lasiodiplodia parva*, (GQ469961.1)¥	100	4	Q	Botryosphaeriaceae (Botryosphaeriales)	*Lasiodiplodia*	C
F4802	S004	*Lasiodiplodia parva*, (GQ469964.1)¥	100	4	Q	Botryosphaeriaceae (Botryosphaeriales)	*Lasiodiplodia*	C
F5068	S009	*Lasiodiplodia theobromae*, (FJ478103.1)¥	99	4	Q	Botryosphaeriaceae (Botryosphaeriales)	*Lasiodiplodia*	C
F4807	S008	*Lasiodiplodia theobromae*, (GQ469934.1)¥	100	4	Q	Botryosphaeriaceae (Botryosphaeriales)	*Lasiodiplodia*	C
F4906	S007	*Lasiodiplodia theobromae*, (GQ469934.1)¥	100	4	Q	Botryosphaeriaceae (Botryosphaeriales)	*Lasiodiplodia*	C
F4904	S006	Fungal sp., (GU566256.1) [63]	99	14	S	Leptosphaeriaceae (Pleosporales)	*Leptosphaeria*	J
F4905	S006	Fungal sp., (GU566256.1) [63]	100	14	S	Leptosphaeriaceae (Pleosporales)	*Leptosphaeria*	J
F4819	S006	Fungal endophyte sp., (EU977287.1) [55]	99	20	D	Lophiostomataceae (Pleosporales)	Unknown	P*
F4831a	S002	Fungal endophyte sp., (EU561602.1)¥	99	19	C	Montagnulaceae (Pleosporales)	*Paraconiothyrium* †	A
F4884	S008	*Penicillium herquei*, (EU833220.1)¥	99	6	O	Trichocomaceae (Eurotiales)	*Penicillium*	V*
F4863	S009	*Penicillium* sp., (JF288548.1) [64]	99	6	O	Trichocomaceae (Eurotiales)	*Penicillium*	T*
F4803	S004	Fungal endophyte sp., (EU561622.1)¥	99	2	J	Amphisphaeriaceae (Xylariales)	*Pestalotiopsis*	G
F4846	S006	Fungal endophyte sp., (EU561622.1)¥	100	2	J	Amphisphaeriaceae (Xylariales)	*Pestalotiopsis*	G
F4878	S008	Fungal endophyte sp., (EU561622.1)¥	100	2	J	Amphisphaeriaceae (Xylariales)	*Pestalotiopsis*	G
F4896	S002	Fungal endophyte sp., (EU561622.1)¥	100	2	J	Amphisphaeriaceae (Xylariales)	*Pestalotiopsis*	G
F4842	S003	*Pestalotiopsis microspora*, (FJ478120.1)¥	99	2	J	Amphisphaeriaceae (Xylariales)	*Pestalotiopsis*	B
F4848	S006	*Pestalotiopsis microspora*, (FJ478120.1)¥	99	2	J	Amphisphaeriaceae (Xylariales)	*Pestalotiopsis*	B
F4872	S004	*Pestalotiopsis* sp., (EU605882.1)¥	99	2	J	Amphisphaeriaceae (Xylariales)	*Pestalotiopsis*	B
F4820	S006	*Pestalotiopsis* sp., [EU605882.1)¥	99	2	J	Amphisphaeriaceae (Xylariales)	*Pestalotiopsis*	B
F4824	S003	*Pestalotiopsis* sp., (EU605882.1)¥	100	2	J	Amphisphaeriaceae (Xylariales)	*Pestalotiopsis*	B
F4829	S005	*Pestalotiopsis* sp., (EU605882.1)¥	100	2	J	Amphisphaeriaceae (Xylariales)	*Pestalotiopsis*	B
F4858	S004	*Pestalotiopsis* sp., (EU605882.1)¥	100	2	J	Amphisphaeriaceae (Xylariales)	*Pestalotiopsis*	B
F4908	S007	*Pestalotiopsis* sp., (EU605882.1)¥	100	2	J	Amphisphaeriaceae (Xylariales)	*Pestalotiopsis*	B
F4857	S002	*Pestalotiopsis* sp., (EU605882.1)¥	99	2	J	Amphisphaeriaceae (Xylariales)	*Pestalotiopsis*	Q*
F4817	S002	*Pestalotiopsis* sp., (HQ832816.1)¥	100	2	J	Amphisphaeriaceae (Xylariales)	*Pestalotiopsis*	B
F4826	S004	*Pestalotiopsis* sp., (HQ832816.1)¥	100	2	J	Amphisphaeriaceae (Xylariales)	*Pestalotiopsis*	B
F4827	S005	*Pestalotiopsis* sp., (HQ832816.1)¥	100	2	J	Amphisphaeriaceae (Xylariales)	*Pestalotiopsis*	B
F4837	S002	*Pestalotiopsis* sp., (HQ832816.1)¥	100	2	J	Amphisphaeriaceae (Xylariales)	*Pestalotiopsis*	B
F4841	S003	*Pestalotiopsis* sp., (HQ832816.1)¥	100	2	J	Amphisphaeriaceae (Xylariales)	*Pestalotiopsis*	B
F4861	S005	*Pestalotiopsis* sp., (HQ832816.1)¥	100	2	J	Amphisphaeriaceae (Xylariales)	*Pestalotiopsis*	B
F4862	S007	*Pestalotiopsis* sp., (HQ832816.1)¥	100	2	J	Amphisphaeriaceae (Xylariales)	*Pestalotiopsis*	B
F4895	S002	*Pestalotiopsis* sp., (HQ832816.1)¥	100	2	J	Amphisphaeriaceae (Xylariales)	*Pestalotiopsis*	B
F4909	S008	*Pestalotiopsis* sp., (HQ832816.1)¥	100	2	J	Amphisphaeriaceae (Xylariales)	*Pestalotiopsis*	B
F5074	S003	*Pestalotiopsis* sp., (HQ832816.1)¥	100	2	J	Amphisphaeriaceae (Xylariales)	*Pestalotiopsis*	B
F4823	S003	*Pestalotiopsis* sp., (HQ832816.1)¥	100	2	J	Amphisphaeriaceae (Xylariales)	*Pestalotiopsis*	B
F4894	S002	*Pestalotiopsis theae*, (HQ832793.1)¥	99	2	J	Amphisphaeriaceae (Xylariales)	*Pestalotiopsis*	G
F4870	S002	Uncultured fungus clone, (JN890176.1) [65]	99	2	J	Amphisphaeriaceae (Xylariales)	*Pestalotiopsis*	G
F4875	S005	Uncultured fungus clone, (JN890258.1) [65]	99	2	J	Amphisphaeriaceae (Xylariales)	*Pestalotiopsis*	B
F4879	S008	Uncultured fungus clone, (JN890258.1) [65]	99	2	J	Amphisphaeriaceae (Xylariales)	*Pestalotiopsis*	B
F5069	S006	Uncultured fungus clone, (JN890258.1) [65]	100	2	J	Amphisphaeriaceae (Xylariales)	*Pestalotiopsis*	B
F5073	S008	Sordariomycetes sp., (JQ760525.1) [65]	99	17	A	Calosphaeriaceae (Calosphaeriales)	*Phaeoacremonium*	L*
F5070	S006	Uncultured fungus clone, (GU721790.1)¥	99	16	T	Cephalotheceae (Sordariales)	*Phialemonium*	F
F5072	S003	Uncultured fungus clone, (GU721790.1)¥	99	16	T	Cephalotheceae (Sordariales)	*Phialemonium*	F
F4886	S004	*Robillarda sessilis*, (HQ608017.1) [58]	99	8	G	Amphisphaeriaceae (Xylariales)	*Robillarda* †	Z*
F4891	S009	*Phoma* sp., (JQ621876.1)¥	99	9	R	Pleosporales†	Unknown	BB*
F4850	S009	Uncultured soil fungus clone, (DQ421180.1) [35]	96	9	R	Unknown	Unknown	M*

OTU codes indicate operational taxonomic units based on 95% sequence similarity. Order and family level identifications were confidently assigned to 82 isolates and genus level identifications were confidently assigned to 80 isolates.

Unpublished strains.

*OTUs occurring only once (singletons).

Tentative phylogenetic placement.

**Table 2:** Bioactivity of sloth hair surface associated fungi isolated on potato dextrose agar (PDA) or 2% malt extract agar (MEA) against causative agents of malaria (*P. falciparum*) and Chagas disease (*T. cruzi*), and against the MCF-7 breast cancer cell line.

Fungus ID	OTU	Isolation Media	Putative Genus	*P. falciparum*	*T. cruzi*	MCF-7
F4813	A	PDA	*Hypocrea*			A
F4814	A	MEA	*Hypocrea*			A
F4815	A	PDA	*Hypocrea*		A	A
F4816	A	PDA	*Hypocrea*		A	A
F4818	D	PDA	*Cytospora*		A	A
F4828	O	PDA	*Fusarium*			A
F4853	A	MEA	*Hypocrea*		-	A
F4854	A	MEA	*Hypocrea*			A
F4855	A	PDA	*Hypocrea*		A	A
F4863	T	PDA	*Penicillium*	A	A	A
F4874	W	MEA	*Bionectria*		A	
F4881	X	MEA	*Fusarium*	A	A	A
F4882	A	MEA	*Hypocrea*		-	A
F4890	K	MEA	*Fusarium*		-	A
F4894	G	MEA	*Pestalotiopsis*			A
F4898	Y	PDA	*Fusarium*		A	A

Bioactive fungi (A) are those causing ≥50% inhibition of growth of parasite or cancer cells in *in vitro* assays.

-  =  extract not tested in this bioassay; empty cell  =  not highly active.

**Table 3:** Bioactivity of fungi from sloth hair isolated on potato dextrose agar (PDA) or malt extract agar (2%; MEA) against a range of Gram-positive and Gram-negative bacteria in the BioMAP assay [30].

				Gram-positive						Gram-negative								
Fungus ID	OTU	Isolation Media	Putative Genus	*B. sub*	*E. fae*	*L. iva*	*S. epi*	*S. au*	MRSA	*Y. pse*	*P. aer*	*S. typ*	*V. chol*	*E. coli*	*A. baum*	*E. aero*	*O. ant*	*P. alc*
F4850	M	PDA	Unknown	A														
F4847	S	MEA	*Arthrinium*												A			
F4821	I	MEA	*Bionectria*	A	A			A	A									
F4806*	R	MEA	*Colletotrichum*	A	A	A	A	A	A									
F4818*	D	PDA	*Cytospora*	A	A	A	A	A	A									
F4839	D	PDA	*Cytospora*	A	A		A	A	A									
F4904	J	PDA	*Leptosphaeria*	A			A	A	A									
F4905	J	PDA	*Leptosphaeria*						A									
F4801	U	MEA	*Endomelanconiopsis*	A														
F4828	O	PDA	*Fusarium*	A	A	A	A	A	A									
F4898*	Y	PDA	*Fusarium*	A	A	A	A	A	A									
F4830	H	MEA	*Hypocrea*						A									
F4807*	C	PDA	*Lasiodiplodia*	A						A		A		WA	A		WA	WA
F4825	C	PDA	*Lasiodiplodia*						A									
F4844*	C	MEA	*Lasiodiplodia*	A	A	A	A	A	A									
F4824	B	MEA	*Pestalotiopsis*						A									
F4829	B	MEA	*Pestalotiopsis*	A			A	A	A									
F4842	B	MEA	*Pestalotiopsis*	A	A		A	A	A									
F4846	G	MEA	*Pestalotiopsis*												A			
F4848	B	MEA	*Pestalotiopsis*												A			

Fungi with particularly potent bioactivity were selected for further study and are marked with an asterisk (*). Fungal extracts causing full cell death are marked ‘A’ and those causing partial cell death are marked ‘WA’.

B. sub  =  Bacillus subtilis 168; E. fae  =  Enterococcus faecium ATCC 6569; L. iva  =  Listeria ivanovii BAA-139; S. epi  =  Staphylococcus epidermis ATCC 14990; S. au  =  Staphylococcus aureus ATCC 29213; MRSA  =  Methicillin Resistant Staphylococcus aureus BAA-44; Y. pse  =  Yersinia pseudotuberculosis IP2666 pIBI; P. aer  =  Pseudomonas aeruginosa ATCC 27835; S. typ  =  Salmonella typhimerium LT2; V. chol  =  Vibrio cholerae O1 (biotype El Tor A1552); E. coli  =  Escherichia coli K12 (BW 25113); A. baum  =  Acinetobacteria baumanii NCIMB 12457; E. aero  =  Enterobacter aerogenes ATCC 35029; O. ant  =  Ochrobactrum anthropi ATCC 49687; P. alc  =  Providencia alcallifaciens ATCC 9886.

### Phylogenetic analyses

The 84 fungal isolates represented 28 operational taxonomic units (OTUs) based on 95% sequence similarity (Table 1). The two most abundant OTUs represented 34 (40.5%) of the isolates. The remaining 50 isolates were represented by 26 distinct OTUs.

BLAST comparisons with GenBank provided preliminary estimations of taxonomic placement and similarity to previously sequenced fungi (Table 1). All isolates were Ascomycota. Fourteen isolates had a top match to uncultured fugal clones and 23 had a top match to cultured but unidentified fungi. The remaining 47 isolates had matches to named strains that tentatively suggested placement in the Sordariomycetes (Xylariales, Glomerellales, Hypocreales), Dothideomycetes (Botryosphaeriales and Pleosporales), and Eurotiomycetes (Eurotiales). To more confidently determine taxonomic placement, sequences were analyzed using maximum likelihood and Bayesian methods.

Sequences that aligned well to one another were partitioned into clusters to create 20 groups of apparently similar species (Table 1; Figure 1). One group contained 29 sequences from sloth-hair fungi, one group contained 20 sequences, and the remaining 18 groups contained between 1 and 6 sequences each. Between 34 and 165 sequences from closely related taxa were compiled for each group to make non-redundant datasets to which one or two appropriate outgroups were added based on literature review.

Following phylogenetic analyses, high level identities (class, order, family) could be confidently assigned to 82 fungi representing 15 families and 10 orders (Table 1; Figure 1). The majority (81.7%) of these were Sordariomycetes. Fungi from this class are well documented sources of bioactive metabolites (e.g., [33] [34]). The remaining isolates were Dothideomycetes (15.9%) and Eurotiomycetes (2.4%).

Eighty isolates could confidently be assigned to 15 genera (Table 1; Figure 1). Two isolates were given more tentative phylogenetic placements. F4886 (Figure 1(G)) was identified as *Robillarda* sp. or as a member of a closely related genus within the Amphisphaeriaceae. Isolate F4831a (Figure 1(C)) was identified as a member of *Paraconiothyrium* sp. or a closely related genus within the Montagnulaceae. The two most common genera, *Pestalotiopsis* sp. and *Hypocrea* sp., were isolated from 7 of 9 and 5 of 9 sloth hair samples, respectively.

Isolate F4891 (Figure 1(R)) did not appear to be closely related to any named sequences in GenBank and could only be identified as possibly a member of the order Pleosporales (Dothideomycetes). Isolate F4850 (Figure 1(R)) had 96% sequence similarity to its closest match in GenBank, an uncultured soil fungus clone (Table 1; [35]). Further analyses are warranted in order to confirm whether isolates F4891 and F4850 are novel fungal species. In general, identifications assigned by phylogenetic analyses closely matched those of the top BLAST hits for each isolate (Table 1).

### Bioactivity of sloth hair isolates

Sloth hair fungi that were cultivated in liquid medium and extracted with ethyl acetate were tested for bioactivity. We considered extracts to be highly bioactive if they caused at least 50% inhibition of the growth (i.e., ≥50% IG) of parasites or cancer cells *in vitro*.

Overall, two of 70 (2.5%) extracts tested were highly active against *P. falciparum*, eight of 62 (12.9%) extracts tested were highly active against *T. cruzi*, and 15 of 73 (20.6%) extracts tested were highly active against the MCF-7 breast cancer cell line (Table 2). Most extracts with bioactivity were active in only one assay (Table 2). Bioactive strains were isolated with equal frequency on MEA and PDA (Table 2). Notably, closely related isolates often differed in bioactivity: for example, three of 20 isolates identified as *Hypocrea* sp. (Table 1) had bioactivity only against MCF-7 breast cancer cells; three were active against MCF-7 breast cancer cells and *T. cruzi* (Table 2); and one was active only against methicillin-resistant *S. aureus* (Table 3).

Of the 16 fungi that had high % IG in at least one *in vitro* assay, 13 belonged to the Hypocreales (Hypocreaceae, Bionectriaceae and Nectriaceae). The remaining three fungi belonged to the Valsaceae (Diaporthales), Amphisphaeriaceae (Xylariales) and Trichocomaceae (Eurotiales). Anti-malarial and anti-cancer activities have been previously reported in the literature from these relatively well-studied fungal lineages (e.g., [36] [37] [38] [39] [40] [41] [42] [43]). However our results represent, to the best of our knowledge, the first reports of anti-trypanosomal activity in *Cytospora* (Valsaceae) and *Bionectria* (Bionectriaceae).

The number of isolates highly bioactive against *T. cruzi* (Table 2) is of particular interest as we so rarely encounter microbes with bioactivity in this assay: only 104 of 2698 fungal endophytes (3.9%) from our overall culture collection are highly bioactive against that parasite [29]. Currently the only treatments available for this illness are nitrofurane and benznidazole, both of which are associated with such toxic side effects that treatment is often abandoned [44].

Of 50 fungal extracts screened by BioMAP, 20 were bioactive against at least one test organism (Table 3). The majority of isolates (16 of 20) active against bacteria in the BioMAP assay belonged to the families Lasiosphaeriaceae (Sordariales), Glomerellaceae (Glomerellales), Valsaceae (Diaporthales), Botryosphaeriaceae (Botryosphaeriales), and Amphisphaeriaceae (Xylariales). The remaining four fungi belonged to the Nectriaceae, Bionectriaceae and Hypocreaceae (Hypocreales). Activity against Gram-positive bacteria was detected far more frequently than against Gram-negative bacteria (Table 3).

Five isolates (*Colletotrichum* sp. F4806 (Figure 1(F)); *Cytospora* sp. 1, strain F4818 (Figure 1(K)); *Fusarium* sp. 1, strain F4898 (Figure 1(M)); *Lasiodiplodia* sp. 1, strain F4807 (Figure 1(Q)); and *Lasiodiplodia* sp. 1, strain F4844 (Figure 1(Q)) had particularly potent activity in the BioMAP assay and were selected for further testing. Serially diluted extracts were re-tested for activity against the panel of 15 bacteria and bioactivity profiles were constructed using normalized MIC values (Table 4).

**Table 4:** Minimum inhibitory concentrations (MIC) of 5 sloth hair associated fungal extracts in the BioMAP antibiotic profile screen [30].

	Minimum Inhibitory Concentration (Normalized 0–1)				
Fungus ID	F4806	F4807	F4818	F4844	F4898
*Bacillus subtilis*	1	0.85	1	1	1
*Staphylococcus epidermis*	0.46	0	0.88	0.87	0.69
*Enterococcus faecium*	0.6	0	0.52	0.6	0.53
*Listeria ivanovii*	0.6	0	0.4	0.46	0.53
*Staphylococcus aureus*	0.87	0	1	0.6	0.85
MRSA	-	-	-	-	*-*
*Yersinia pseudotuberculosis*	0	0.69	0	0	0
*Pseudomonas aeruginosa*	0	0	0	0	0
*Salmonella typhimerium*	0	0.53	0	0	0
*Vibrio cholerae*	0	0	0	0	0
*Escherichia coli*	0	0.69	0	0	0
*Acinetobacter baumanii*	0	1	0	0	0
*Enterobacter aerogenes*	0	0	0	0	0
*Ochrobactrum anthropi*	0	0.69	0	0	0
*Providencia alcalifaciens*	0	0.53	0	0	0

To generate a concentration- independent bioactivity profile, raw assay results were normalized giving a range of values from 0 (inactive) to 1 (most bioactive). These bioactivity fingerprints were then compared to fingerprints of known antibiotics from all the major structural classes, which had previously been tested in the BioMAP assay. The bioactivity fingerprint of F4807 was of particular interest as it did not match that of any antibiotic previously tested in the BioMAP screen.

-  =  Test failed, data not available.

The extract from *Lasiodiplodia* sp. 1 (strain F4807) was of particular interest as it had potent and specific activity against Gram-negative bacteria (Table 4). The bioactivity profile of the isolate did not match that of any of the known antibiotic classes previously tested in the BioMAP assay, suggesting a potentially novel mode of action (see [30]). Infections caused by emerging multidrug-resistant (MDR) Gram-negative bacteria are becoming a major clinical concern worldwide [45]. These infections most commonly include MDR *Pseudomonas aeruginosa*, extended-spectrum β-lactamase producing Enterobacteriaceae, and MDR *Acinetobacter baumannii* [46]. Recent data suggest that infections traced to *Staphylococcus aureus* bloodstream isolates are in decline, but that infections by *Escherichia coli*, the most common Gram-negative species responsible for infections in human blood isolates, are increasing in frequency [47]. That report [47] also highlights the paucity of antibacterial agents under development for Gram-negative bacteria, with most effort being focused on Gram-positive MRSA. The increasing prevalence of MDR organisms is mainly due to overuse of broad-spectrum antibiotics and poor antibiotic stewardship [48]. Hence the specificity of the extract from strain F4807 for Gram-negative strains is highly valuable and worth pursuing for further analysis.

Fungi isolated in this study were taxonomically consistent with groups of fungi known to occur in soil and in plants as pathogens, saprotrophs, or endophytes [11] [49]. Sloths may encounter such fungi incidentally from air spora, or via direct contact when they descend from trees in order to defecate and urinate, at which time they dig a hole in the soil that they subsequently cover with leaf litter [50]. Strikingly, comparison with a large collection of endophytes from terrestrial plants in Panama ([29]; 1269 strains) revealed that sequences from 29 isolates from sloth hair were identical to strains obtained from plants (100% similarity over the full sequence length). Thus this study extends the known host range and ecological mode of putative endophytes. Some of these fungi may affiliate directly with the green alga that inhabits coarse hair of sloths, much like endolichenic fungi associate with green algae in lichen thalli and are often taxonomically similar to endophytes in the same environments [51] [52].

Potential roles of these fungi in the health of sloths have not been investigated. Araújo Xavier *et al*. [50] reported fungal infections in *B. variegatus* caused by the fungal pathogen *Microsporum* (which also causes dermatitis in humans; [53]), but this genus was not recovered in the present study or in previous work by Suutari *et al*. [16]. In contrast to the human microbiome, where fungi comprise <0.01% of microbial communites on external surfaces such as skin [54], Suutari *et al*. [16] reported that fungi represented 8% of the flora in surveys of sloth-hair microbes. The high abundance and diversity of fungi associated with sloth hair, coupled with their bioactivity, may speak to a biological importance to sloths that is yet unexplored.

The pressing need for new medications continues to represent one of humanity's greatest challenges. It is commonly agreed that the vast majority of bioactive microbes remain to be discovered [9] and newly discovered microbial taxa hold an important promise of novel chemistry. Abundant evidence points to novel environments as promising sources of as yet undescribed microorganisms [55]. Here we have demonstrated that hair of the three-toed sloth (*Bradypus variegatus*) in Panama is a rich source of diverse bioactive fungi. Strains isolated here included members of some well-studied lineages (e.g., Hypocreales; Pleosporales), but also members of understudied or potentially novel groups (e.g., *Endomelanconiopsis* sp. F4801; *Robillarda* sp. F4886; Ascomycota sp. F4891; Ascomycota sp. F4850). We anticipate that additional novel taxa could be found on sloth hair by expanding survey methods to diverse types of media and using high-throughput methods to assess community composition. We also anticipate that different taxa may be isolated from sloths in other regions, much as communities of endophytes in tropical plants differ markedly at a regional scale [56] [57]. Thus our work suggests that fruitful exploration of the sloth microbiota is warranted for potential applications in drug discovery.

## Supporting Information

Table S1 **Sloth hair fungus ID and Genbank accession numbers.**
(DOCX)
