# Correction: Sloth Hair as a Novel Source of Fungi with Potent Anti-Parasitic, Anti-Cancer and Anti-Bacterial Bioactivity

**DOI:** 10.1371/annotation/10c47668-8c49-48d2-ba65-60420af463b6

**Published:** 2014-01-30

**Authors:** Sarah Higginbotham, Weng Ruh Wong, Roger G. Linington, Carmenza Spadafora, Liliana Iturrado, A. Elizabeth Arnold

Figure 1 was published in an incomplete form. Please see all sections of Figure 1 here:

Figure 1A - 1D: http://www.plosone.org/corrections/pone.0084549.g001.1.cn.tif

Figure 1E - 1H: http://www.plosone.org/corrections/pone.0084549.g001.2.cn.tif

Figure 1I: http://www.plosone.org/corrections/pone.0084549.g001.3.cn.tif

Figure 1J: http://www.plosone.org/corrections/pone.0084549.g001.4.cn.tif

Figure 1K - 1N: http://www.plosone.org/corrections/pone.0084549.g001.5.cn.tif

Figure 1O - 1P: http://www.plosone.org/corrections/pone.0084549.g001.6.cn.tif

Figure 1Q - 1T: http://www.plosone.org/corrections/pone.0084549.g001.7.cn.tif

