# Supplementary material for: Sloth Hair as a Novel Source of Fungi with Potent Anti-Parasitic, Anti-Cancer and Anti-Bacterial Bioactivity
Source: PLoS One. 2014 Jan 15;9(1):e84549. doi: 10.1371/journal.pone.0084549 (PMC3893167; doi:10.1371/journal.pone.0084549)
Supplement: Table S1 — Sloth hair fungus ID and Genbank accession numbers. (DOCX) [file pone.0084549.s001.docx]

**Table S1**: Sloth hair fungus ID and Genbank accession numbers

| **Fungus ID** | **Accession Number** |
| --- | --- |
| F4801 | KF746076 |
| F4802 | KF746077 |
| F4803 | KF746078 |
| F4806 | KF746079 |
| F4807 | KF746080 |
| F4812 | KF746081 |
| F4813 | KF746082 |
| F4814 | KF746083 |
| F4815 | KF746084 |
| F4816 | KF746085 |
| F4817 | KF746086 |
| F4818 | KF746087 |
| F4819 | KF746088 |
| F4820 | KF746089 |
| F4821 | KF746090 |
| F4823 | KF746091 |
| F4824 | KF746092 |
| F4825 | KF746093 |
| F4826 | KF746094 |
| F4827 | KF746095 |
| F4828 | KF746096 |
| F4829 | KF746097 |
| F4830 | KF746098 |
| F4831 | KF746100 |
| F4831a | KF746099 |
| F4837 | KF746101 |
| F4839 | KF746102 |
| F4841 | KF746103 |
| F4842 | KF746104 |
| F4844 | KF746105 |
| F4845 | KF746106 |
| F4846 | KF746107 |
| F4847 | KF746108 |
| F4848 | KF746109 |
| F4850 | KF746110 |
| F4852 | KF746111 |
| F4853 | KF746112 |
| F4854 | KF746113 |
| F4855 | KF746114 |
| F4856 | KF746115 |
| F4857 | KF746116 |
| F4858 | KF746117 |
| F4860 | KF746118 |
| F4861 | KF746119 |
| F4862 | KF746120 |
| F4863 | KF746121 |
| F4870 | KF746122 |
| F4872 | KF746123 |
| F4873 | KF746124 |
| F4874 | KF746125 |
| F4875 | KF746126 |
| F4876 | KF746127 |
| F4877 | KF746128 |
| F4878 | KF746129 |
| F4879 | KF746130 |
| F4881 | KF746131 |
| F4882 | KF746132 |
| F4883 | KF746133 |
| F4884 | KF746134 |
| F4886 | KF746135 |
| F4889 | KF746136 |
| F4890 | KF746137 |
| F4891 | KF746138 |
| F4894 | KF746139 |
| F4895 | KF746140 |
| F4896 | KF746141 |
| F4897 | KF746142 |
| F4898 | KF746143 |
| F4900 | KF746144 |
| F4901 | KF746145 |
| F4902 | KF746146 |
| F4904 | KF746147 |
| F4905 | KF746148 |
| F4906 | KF746149 |
| F4908 | KF746150 |
| F4909 | KF746151 |
| F4913 | KF746152 |
| F5068 | KF746153 |
| F5069 | KF746154 |
| F5070 | KF746155 |
| F5071 | KF746156 |
| F5072 | KF746157 |
| F5073 | KF746158 |
| F5074 | KF746159 |
